# Analysis of atherosclerotic plaque distribution in the carotid artery

**DOI:** 10.1002/clc.23903

**Published:** 2022-09-10

**Authors:** Shin‐Seok Yang, Shin‐Young Woo, Dong‐Ik Kim

**Affiliations:** ^1^ Division of Vascular Surgery, Department of Surgery, Samsung Medical Center Sungkyunkwan University School of Medicine Seoul Korea

**Keywords:** atherosclerotic plaque, carotid artery stenosis, Duplex scan, shear stress

## Abstract

**Background:**

The present study was designed to investigate the hypothesis that the outer wall at the carotid bifurcation is the most common area of atherosclerotic plaque deposition due to the low shear stress.

**Hypothesis:**

We hypothesized that the most common site of arteriosclerosis in carotid arteries is different in the early and late stages.

**Methods:**

This is an observational study of patients with <50% stenosis of the common and internal carotid arteries (ICAs) identified by Duplex ultrasound in our health promotion center. Plaque location was categorized as a quarter of the cross‐section in the distal common carotid artery (CCA) and proximal ICA. Carotid plaque score (CPS) was calculated by the addition of one point for each detected section. The sum of CPSs was calculated for each section.

**Results:**

Among 3996 Duplex scans of carotid arteries in 999 patients between June 2020 and October 2020, a total of 569 patients (73.6% male; mean age, 68.4± 9.1 years; 652 CCAs and 567 ICAs) were included. Total CPS was high in the anterior and posterior sections. The distribution in the ICA was: 308 (31.0%) anterior, 90 (9.0%) medial, 373 (37.5%) posterior, and 224 (22.5%) lateral section. The distribution in the CCA was 385 (32.6%) anterior, 103 (8.7%) medial, 528 (44.7%) posterior, and 165 (14.0%) lateral section. The axial distribution of posterior and lateral sections was significantly different according to the directional flow (*p* < .001).

**Conclusions:**

Anterior and posterior sections of the CCA and ICA were atherosclerotic plaque‐prone sites. This result is different from the tendency of atherogenesis to affect the lateral section having low shear stress at the carotid bifurcation.

## 1 INTRODUCTION

Poiseuille's law is used to estimate arterial wall shear stress from blood flow velocity, viscosity, and arterial radius. Shear stress actively contributes to the formation of atherosclerosis and arterial plaques.^1^ Low wall shear stress increases vascular endothelial permeability and induces monocyte infiltration into the arterial wall leading to the migration of smooth muscle cells into the subintimal layer and progression of local atherosclerosis.^2^

Arterial plaques preferentially develop in the outer walls of arterial bifurcations as these are points of blood flow recirculation and stasis due to shear stress decrease.^3^ Therefore, atherosclerotic plaques primarily occur at major arterial bifurcations, such as the inner wall curvature of the aortic arch and aortoiliac bifurcation. The outer walls of carotid bifurcations represent a common site of atherosclerotic plaques.^4^

The Duplex scan is a widely used imaging modality to evaluate the degree of arterial stenosis and luminal narrowing using B‐mode and spectral wave Doppler. Because of carotid artery stenosis severity and its association with the risk of stroke, this risk can be estimated by Doppler‐derived velocity.^5^ Another merit of a Duplex scan is the ease of early detection and quantification of the characteristics of atherosclerotic plaques using B‐mode real‐time ultrasound.^6^ The carotid Duplex allows physicians to visualize the extent of arterial wall and lumen surface involvement. In particular, B‐mode ultrasound has been considered the modality of choice for the evaluation of carotid intima‐media thickness and plaque echotextures.^7^

A previous study suggested that carotid plaques are more common at the carotid bifurcation due to transient reverse flow in patients with symptomatic stenosis.^8^ However, the anatomic distribution of carotid plaques in patients with mild carotid stenosis remains unclear. Therefore, we performed this study to assess the anatomical site and extent of common and internal carotid atherosclerotic plaque deposition using Duplex carotid scans in patients with a low degree of carotid stenosis.

## 2 METHODS

### 2.1 Study design

Between June 2020 and October 2020, carotid artery Duplex scans were performed in 1036 patients who were referred to our health promotion center for carotid stenosis screening. We reviewed patient medical records and collected demographic and comorbidity data. The study was approved by the local institutional review board (IRB File No. 2020‐08‐030). Informed consent was waived because of the retrospective nature of the study and the analysis used anonymous clinical data. This study was not registered in the ClinicalTrials.gov database.

### 2.2 Study inclusion and exclusion criteria

We enrolled patients >40 years old with mild common and/or internal carotid stenosis (<50% degree of stenosis). We excluded those with ipsilateral Duplex scans who underwent carotid endarterectomy or stenting.

### 2.3 Carotid artery Duplex imaging

**Figure 1:**
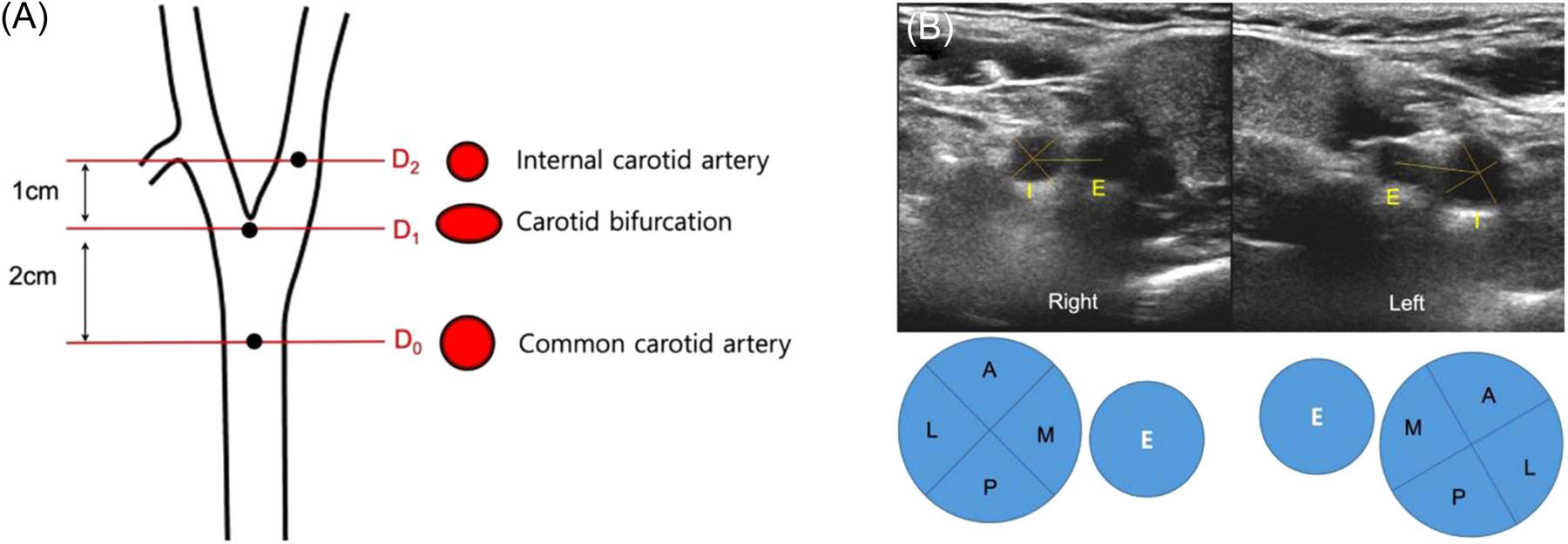
(A) Anatomical landmarks for the Duplex scan. (B) Sectional classification of the internal carotid artery (I) according to the position of the external carotid artery (E). Medial section (M), inner 90° wall of flow divider; lateral section (L) outer 90° wall of flow divider; anterior section (A), superficial 90° wall between medial and lateral section; and posterior section (P), deep 90° wall between lateral and medial section.

Duplex scans were performed by six Registered Vascular Technologists® using ultrasound devices with probes (iU22; Philips; LOGIQ™ E9 XDclear GE Medical Systems). Results were reviewed by two vascular surgeons. The degree of carotid stenosis was estimated as normal, stenosis <50%, stenosis 50%–70%, stenosis >70%, and total occlusion according to the Society of Radiologists in Ultrasound consensus.^5^ Patients were scanned in the supine position with contralateral rotation of the head in a darkened examination room. Peak systolic, end‐diastolic, and mean blood flow velocities were recorded with the sample volume placed in the center of carotid arteries. Axial and longitudinal grayscale ultrasound images of bilateral carotid arteries were recorded in the common carotid (*D*_0_, 2 cm below the bifurcation), at the carotid bifurcation (*D*_1_), and in the proximal internal carotid artery (ICA; *D*_2_, 1 cm above the bifurcation) and described the location of carotid plaques using standardized equipment presets and image acquisition protocol (Figure 1A).

### 2.4 Data collection and statistical analysis

Duplex scans were performed on all patients at the health promotion center once during the study period. Carotid plaque presence was defined as a focal wall thickness greater than 1.5 mm or a focal thickening greater than 50% of the adjacent wall segments. To evaluate the distribution of carotid atherosclerotic plaques, we analyzed the circumferential and axial distribution on Duplex ultrasound axial images. We classified the location of plaques as anterior, posterior, medial, and lateral sections from the grayscale axial image of the common carotid, at the bifurcation, and in the ICA (Figure 1B). Carotid plaque score (CPS) was calculated by the addition of one point for each detected section. In cases of multisectional involvement, we scored all involved sections. The *χ* ^2^ analysis was used to compare the axial distribution change of CPS. Two‐tailed *p* < .05 were considered statistically significant.

## 3 RESULTS

Among 3996 common and ICAs in 999 patients, a total of 2777 arteries were excluded because of stenosis of more than 50% (*n* = 351), previous performance of carotid endarterectomy (*n* = 178), previous insertion of carotid artery stent (*n* = 20), young age (<40 years old) (*n* = 144), and no presence of carotid plaques (*n* = 2084). The remaining 1219 carotid arteries (652 common carotid and 567 internal carotids) in 569 patients with mild carotid plaque distributions were enrolled in the study (Supporting Information File).

The mean age was 68.4 ± 9.1 years and 419 (73.6%) patients were male. Age in the 60s was most common. Table 1 shows the demographics of the population.

**Table 1:** Patient demographics

Variables	*n* (%)
Age (years)	
Median (IQR, range)	68 (62, 75, 40–96)
Mean ± SD	68.4 ± 9.1
40–49	14 (2.5)
50–59	75 (13.2)
60–69	224 (39.4)
70–79	189 (33.2)
≥80	67 (11.8)
Comorbidities	
Hypertension	227 (39.9)
Diabetes mellitus	152 (26.7)
Coronary artery disease	167 (29.3)
Hyperlipidemia	311 (54.7)
Cerebrovascular disease	53 (9.3)
Medications	
Antiplatelet	496 (87.2)
Antilipidemic	505 (88.8)
Anticoagulant	134 (23.6)
Antihyperglycemic	201 (35.3)

Abbreviation: IQR, interquartile range.

Of 569 patients, 567 ICAs (273 right and 294 left) were analyzed. Single‐section distributions were detected in 266 (46.9%) ICAs. The CPSs of ICAs were 308 anterior, 90 medial, 373 posterior, and 224 lateral sections. The circumferential distribution of ICA plaques is shown in Table 2.

**Table 2:** Total carotid plaque scores of internal carotid artries (*n* = 567)

Section	Single distribution (*n* = 266)	Multiple distribution (*n* = 301)	Total
Anterior	97	211	308 (31.0%)
Medial	11	79	90 (9.0%)
Posterior	121	252	373 (37.5%)
Lateral	37	187	224 (22.5%)
Total score	266	729	995 (100%)

Common carotid plaques were evaluated in 652 common carotid arteries (CCAs) (334 right and 318 left). Of these, 319 CCAs (48.0%) had single plaque distributions. The CPSs of CCAs were similar to those of ICAs (385 anterior, 103 medial, 528 posterior, and 165 lateral sections). Table 3 shows the plaque distributions in CCAs.

**Table 3:** Total carotid plaque scores of common carotid arteries (*n* = 652)

Section	Single distribution (*n* = 319)	Multiple distribution (*n* = 333)	Total
Anterior	85	300	385 (32.6%)
Medial	5	97	103 (8.7%)
Posterior	216	312	528 (44.7%)
Lateral	13	152	165 (14.0%)
Total score	319	862	1181 (100%)

The sectional distribution of plaques was analyzed along the axial sequence from CCA to ICA. The plaque distribution in anterior and medial sections was similar in CCAs and ICAs. However, there were significant differences in the sectional distribution of posterior and lateral plaques according to the axial direction of blood flow (Table 4).

**Table 4:** Axial distribution change of carotid plaque score

Section	CCA (*n* = 652)	ICA (*n* = 567)	*p*‐Value*
Anterior	385 (32.6%)	308 (31.0%)	.412
Medial	103 (8.7%)	90 (9.0%)	.791
Posterior	528 (44.7%)	373 (37.5%)	.001
Lateral	165 (14.0%)	224 (22.5%)	<.001
Total score	1181 (100%)	995 (100%)	

Abbreviations: CCA, common carotid artery; ICA, internal carotid artery

* *χ* ^2^ test.

## 4 DISCUSSION

The relationship between atherosclerotic plaque distribution and hemodynamic shear stress in vessel wall remodeling is well established. In previous studies, the distribution of atherogenesis was preferentially reported in the outer vessel walls at blood flow bifurcations and points of flow recirculation.^9^, ^10^, ^11^ Atherogenesis at the carotid bifurcation is a key factor in the contribution of internal carotid stenosis to distal embolic infarction.^12^ Previous studies were focused on the relationship between the risk factors of atherosclerosis and carotid stenosis and the need for revascularization to prevent embolic stroke.^13^, ^14^, ^15^

This study was designed to investigate the pattern of early‐stage atherogenesis at the carotid bifurcation. The hemodynamic flow analysis and corresponding pathological sections from carotid autopsy specimens demonstrated that the greatest plaque distribution occurred in the outer wall of the carotid bulb where low shear stress and blood flow stasis contribute to flow direction reversal.^16^, ^17^, ^18^ Previous reports showed flow dividers at the carotid bifurcation in the late stage of atherosclerosis. In this study, we found that atherosclerotic plaques at the carotid bifurcation in the early stage are preferentially localized in the anterior and posterior sections.

Axial distribution of plaques at the carotid bifurcation is another major interest of this study. Atherosclerotic plaques primarily develop in the zone of low shear stress. This zone is an outer vessel wall at bifurcations with sparing flow dividers and at the inner wall of curvatures such as the aortic bifurcation or aortic arch.^19^ Pulsatile arterial blood flow in the normal carotid bulb has two distinct components of flow direction; these consist of laminar flow near the flow bifurcation and transient reverse flow in a zone of the posterolateral aspect.^20^ Therefore, helical pulsatile flow generated in the carotid bulb contributes to the alteration of shear stress in the proximal ICA. However, little is known of the progressive pattern of atherogenesis at the carotid bifurcation. Our study shows the early distribution of atherosclerotic plaques in the carotid bulb including the distal CCA and proximal ICA. We found that the axial distribution of plaques tends to progress to the lateral section and regress to the posterior section. We infer that the atherosclerosis of the outer area progresses faster with carotid stenosis. Further research will be required to confirm the relationship between the progression of mild and moderate stenosis to severe stenosis.

Atherosclerotic plaque instability is a main pathogenetic mechanism of clinical syndromes including acute coronary syndrome (ACS) and acute ischemic stroke syndrome in patients with cardiovascular disease. Atherosclerosis is an inflammatory response to various cardiovascular risk factors, hemodynamic factors, and toxins, resulting in the increase of endothelial permeability, deposition of extracellular lipid particles and inflammatory cells, and plaque formation. Injury to the fibrous cap which envelops the plaque core that includes a lipid matrix and macrophage accumulation results in platelet activation and thrombus formation. Plaque rupture and erosion of atherosclerotic plaques propagate distal thromboembolic events such as ACS or stroke.^21^, ^22^ Carotid atherosclerosis is involved in 15%–20% of ischemic strokes.^23^ In particular, type 2 diabetes mellitus combined with carotid atherosclerosis is associated with a higher risk of ischemic stroke in the general population and in patients who underwent carotid revascularization.^24^, ^25^ For these patients, modulation of the overinflammatory reaction applied to stabilize the atherosclerotic plaque is required. D'Onofrio et al.^26^ demonstrated the effect of anti‐inflammatory modulation using a sodium‐glucose co‐transporter 2 inhibitor (SGLT2i) in cases of diabetic atherosclerotic carotid plaques. The authors concluded that the SGLT2i suppresses the inflammatory process associated with carotid plaques in diabetic patients who underwent carotid endarterectomy. These findings suggest the effectiveness of SGLT2i in establishing favorable inflammatory modulation and plaque stabilization.^26^ In addition, microRNA modulation to suppress inflammatory biomarkers in prediabetic and nondiabetic patients was associated with a reduction of major adverse cardiac events in asymptomatic severe carotid stenosis.^27^ Further studies are needed to determine whether inflammatory modulation is effective in the stabilization of early‐stage atherosclerotic plaques.^28^

This study has several limitations. Patients were recruited in a healthcare promotion setting in which repeat scans were not possible. Therefore, the result was our inability to confirm changes in plaque distribution over time. An additional limitation was a lack of consideration of individual geometric anatomy at the carotid bifurcation. A Duplex scan is an excellent diagnostic tool for evaluating stenosis and plaque distribution, but evaluation of flow divider angle at the bifurcation is difficult by this method. Therefore, we did not calculate the shear stress through an individualized geometric model in this study. In addition, we did not identify relationships between comorbidities and plaque distribution changes in our subanalysis. However, because the increase of plaque instability due to flow direction and shear stress in the lateral section is the main pathogenesis of thromboembolic attacks in patients with more than >50% carotid artery stenosis, our results can be indirect evidence for low stroke risk in the early stages of carotid artery stenosis. Finally, intra‐ and interobserver variability in circumferential and axial plaque distribution determination was not assessed.

## 5 CONCLUSION

The results of this study indicate that atherosclerotic plaques in cases of mild carotid stenosis develop most often in anterior and posterior sections of the common and ICAs. In axial distribution, lateral section plaque development is increased at the entrance to the ICA. This result is not consistent with the tendency of carotid plaques to affect the lateral section, the low shear stress area of the carotid bifurcation. Further validation of the association between low shear stress and distribution of early atherosclerotic plaques in the carotid artery is needed.

## CONFLICT OF INTEREST

The authors declare no conflict of interest.

## Supporting information

Flow chart showing the number of patients.

## Data Availability

Due to its proprietary nature and ethical concerns, supporting data cannot be made openly available.
