# Supplementary figures and images for: Analysis of atherosclerotic plaque distribution in the carotid artery
Source: Clin Cardiol. 2022 Sep 10;45(12):1272–6. doi: 10.1002/clc.23903 (PMC9748747; doi:10.1002/clc.23903)

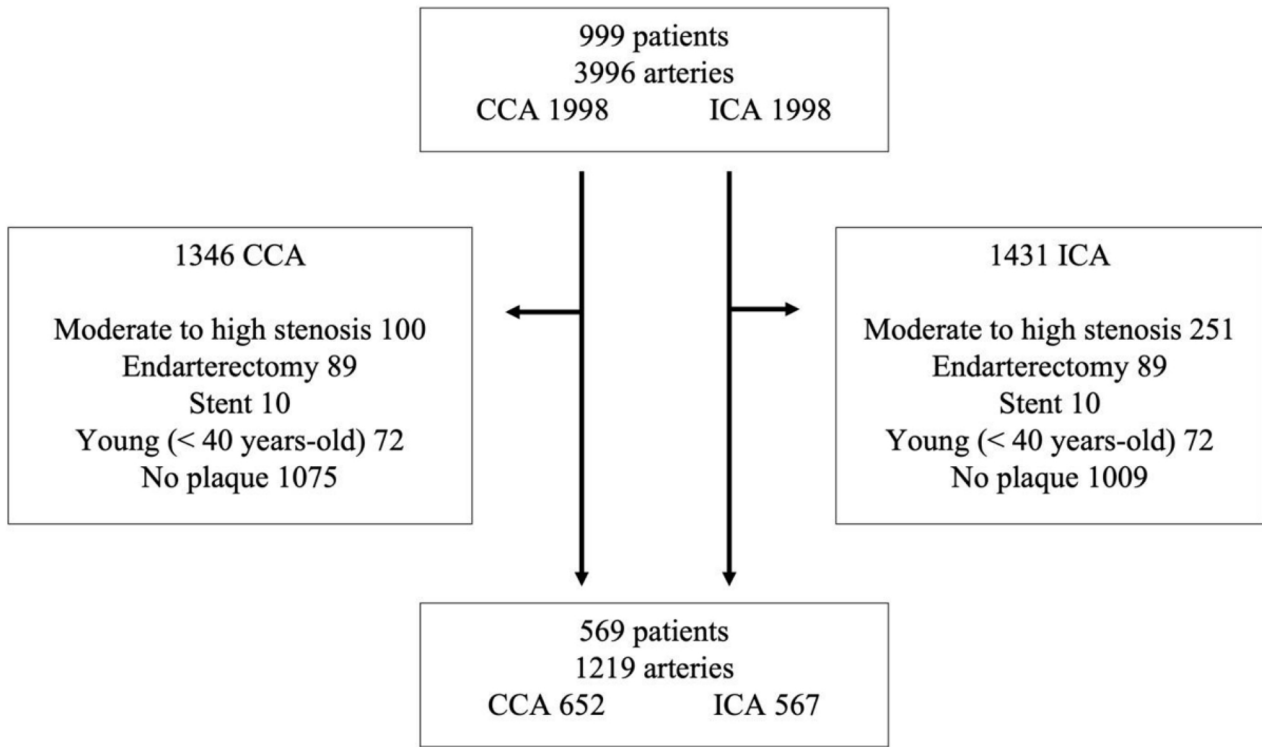

CCA, common carotid artery; ICA, internal carotid artery

Supplement: Supplementary file 1 — Flow chart showing the number of patients. [file CLC-45-1272-s001.pdf]
